# Mechanism of retinoic acid-induced transcription: histone code, DNA oxidation and formation of chromatin loops

**DOI:** 10.1093/nar/gku958

**Published:** 2014-10-15

**Authors:** C. Zuchegna, F. Aceto, A. Bertoni, A. Romano, B. Perillo, P. Laccetti, M. E. Gottesman, E. V. Avvedimento, A. Porcellini

Nucleic Acids Res. 2014 42 (17): 11040-55. doi: 10.1093/nar/gku823

The authors have forgotten to acknowledge funding from Epigenomics Flagship Project—EPIGEN, C.N.R. in their article. The full list of funding agencies and institutions is given below.

## FUNDING

University Federico II of Naples [PhD fellowship to C.Z. and A.R.]; P.O.R. Campania FSE 2007–2013 Project CREMe [CUP B25B09000050007 to C.Z.]; AIRC IG [11364 to V.E.A.]; Fondazione Medicina Molecolare e Terapia Cellulare, Universita’ Politecnica delle Marche [to V.E.A.]; **Epigenomics Flagship Project—EPIGEN, C.N.R.** Funding for open access charge: Epigenomics Flagship Project—EPIGEN, C.N.R.

